# Impact of the COVID-19 Lockdown on Children's Psychosocial Well-Being: A Cross-Sectional Study in Saudi Arabia

**DOI:** 10.7759/cureus.39902

**Published:** 2023-06-03

**Authors:** Abeer M AlHarbi, Abdulrahman A Alghamdi, Jawad M Alabbasi, Nawaf I Alsufyani, Ahmed A Alharbe, Saleh M Abuaunouq

**Affiliations:** 1 Pediatric Medicine, King Abdulaziz Medical City Riyadh, Riyadh, SAU; 2 College of Medicine, King Saud Bin Abdulaziz University for Health Sciences, Riyadh, SAU

**Keywords:** covid 19 impact of lockdown, saudi arabia, lifestyle, children, pandemic, quarantine, covid-19

## Abstract

Background

The impact of COVID-19 on children is a vital topic to cover since the quarantine lasted for months, and limited research explored this effect locally in Arab countries. We studied the impact of the COVID-19 lockdown on the psychosocial well-being of children aged 1-18 years who were living in Saudi Arabia during the time of the pandemic.

Method

A total of 387 participants' responses were collected using online questionnaires (valid and reliable) composed of three sections with open and close-ended questions by the child's legal guardians. This cross-sectional study was conducted in Saudi Arabia and targeted children aged 1-18 years of both genders using a convenience sampling technique. One questionnaire assessed the child's behavior and sleep pattern, while the other evaluated child's activity and social skills. We analyzed the data using Statistical Package for Social Sciences (SPSS) version 20.0 (IBM Corp., Armonk, NY).

Results

Half of the children were 1-6 years (196; 50.6%), and the caregivers of more than half (225; 58.2%) were mothers. Two-thirds (234; 60.5%) of the children were male. Apart from a poor appetite for food and eating (non-nutritional) junk food, which was not significant (p-value > 0.05), all other factors, behavior, sleep patterns, activity, and social skills, all other factors were significantly affected by COVID-19 (p<0.05).

Conclusion

This study found that the COVID-19 pandemic had a negative impact on children's psychosocial well-being. It is recommended to implement actions that aim to enhance the ability of children to cope with challenges.

## Introduction

COVID-19, also known as coronavirus, originated in Wuhan City, Hubei Province of China, and is believed to have been contacted at first through consuming animals and seafood from the animal wet market in Wuhan [1]. To date, the World Health Organization has reported 765,903,278 confirmed cases of COVID-19 worldwide and 6,927,378 deaths [2]. The total number of confirmed cases in Saudi Arabia is 841,469, and the death toll is 9,646 [3]. Although the mortality rate for this disease is almost 2.7%, its transmission poses a problem that needs a countermeasure [4]. As a result, lockdowns have been enforced in many countries, and Saudi Arabia had one in March 2020 that continued until June 2020. This sudden change impacted the lifestyles of many, and even though some people benefited from the lockdown period by doing valuable things like working on their hobbies or education, many others had a hard time mentally and physically because they could not get out of their homes and do their daily tasks [5]. Those who are more susceptible, like children, have been shown to be affected; a study from Bangladesh reported that "large proportions of children are suffering from mental health disturbances in Bangladesh during the lockdown period" [6].

It is not the first time that lockdowns have been implemented due to pandemics. A quarantine was first done in the 14th century to protect coastal cities from plague epidemics, and it was also done for many other diseases like cholera in the 19th century. Other diseases, such as diphtheria and viral hemorrhagic fevers (such as Marburg, Ebola, and Crimean-Congo), also forced governments to force lockdowns [7]. When you look at the epidemics in the past century, COVID-19 is the most dangerous of them all because of its fast spread and associated symptoms. This has affected adults and children alike and created uncertainty in many communities worldwide. Stress among parents became common during such an uncertain situation which will, in turn, reflect on their offspring [8].

Children need a sense of security in order to grow up healthy. However, COVID-19 has caused distress that may impact a child's psychological well-being. The objective of this study was to assess the change of lifestyle among children during the pandemic and highlight how the lockdown impacted the children's psychosocial well-being and evaluate the most common behavioral alterations before and during the lockdown.

## Materials and methods

Study population

This was an observational comparative cross-sectional study design; data was collected using a convenience sampling technique. To determine the sample size, we obtained the population number of children living in Saudi Arabia from the Saudi General Authority for Statistics, which was 107,499,44 [9]. We then used Raosoft® software (Raosoft Inc., Seattle, WA) available on their website (http://www.raosoft.com/samplesize.html) which provided a sample size of 385 using a margin of error of 5% with a 95% confidence level. The inclusion criteria were parents/caregivers of children aged 1-18 years living in Saudi Arabia, irrespective of gender. 

Data collection instrument (questionnaire)

The study's objective was to evaluate the parents' perspective on their children's psychosocial well-being and lifestyle before and during COVID-19. The data was collected using two different questionnaires followed by demographic questions. The first questionnaire comprised six questions assessing behavior and sleep pattern. The second questionnaire had 10 questions evaluating the child's activity and social skills. Both the questionnaires used a Likert scale of 0 for "it did not happen" to 3 for "happened all the time". The Likert scale was taken from Kessler psychological distress scale [10]. The questionnaire was both in English and Arabic languages. 

Validity and reliability

For content validity, two independent subject experts were included to review the questionnaire and give feedback. Face validity was carried out by medical educationists. When conducting the pilot study among a smaller population, we got feedback from the fillers and calculated Cronbach's alpha (internal reliability). The questions were modified accordingly, and Cronbach's alpha was calculated as 0.809, which was considered very good. 

Data collection method and ethical considerations

This study was approved by the ethics committee at King Abdullah International Medical Research Center (KAIMRC). Data were collected using Google Forms; the survey was distributed through social media (WhatsApp and Twitter). An informed consent form was attached to the questionnaire in which the aim of the study, the confidentiality of the participants, and the right to withdraw from the study at any time without penalty were explained at the beginning of the questionnaire, and agreeing to participate was taken as consent. The data was kept in a secured computer with password protection. Only the research team members had access to the data. No names or identifiers were collected to maintain confidentiality. 

Statistical analysis

The Excel file was downloaded from the Google Form website, and the data were then transferred to IBM SPSS version 20.0 for analysis. Categorical variables were presented as frequencies and percentages. The questions regarding the children's psychosocial well-being and lifestyle were presented as the median and interquartile range (IQR). Wilcoxon signed-ranks test was used to compare children's well-being and lifestyle scores before and during COVID-19. P-value <0.05 was considered significant.

## Results

A total of 387 participants/caregivers responded. Among the caregivers, more than half (225; 58.2%), were mothers. Half of the caregivers' children (196; 50.6%), belonged to the 1-6 years age group. Nearly two-thirds of the respondents were males (234; 60.5%), and most children's orders were in the 1st-4th category. Only 28 (7.2%) out of 387 had a chronic illness. For the region of residence, 265 (68.5%) were living in the Central region, and most of the respondents (365; 89.4%), were married at the time of the survey. The rest of the demographic information can be visualized in Tables 1-2.

**Table 1:** Demographics of the participants/caregivers n=Number

Occupation		n=387	%
Relation			
	Father	76	19.6
	Mother	225	58.2
	Other	86	22.2
Child's Age			
	1-6 years	196	50.6
	7-12 years	107	27.7
	13-18 years	84	21.7
Gender			
	Male	234	60.5
	Female	153	39.5
Child's Order			
	1st-4th	269	69.5
	5th-9th	43	11.1
	10th-14th	3	0.8
	15th-last	72	18.6
Chronic illness			
	No	359	92.8
	Yes	28	7.2
Region of Residence			
	Central region	265	68.5
	Southern region	53	13.7
	Western region	47	12.1
	Eastern region	17	4.4
	Northern region	5	1.3
Marital Status			
	Married	346	89.4
	Divorced	21	5.4
	Widowed	4	1
	Other	16	4.2

**Table 2:** Social and occupational information of the caregivers n=number

Occupation		n=387	%
Administrative Job			
	No	324	83.7
	Yes	63	16.3
Military Job			
	No	347	89.7
	Yes	40	10.3
Educational Job			
	No	301	77.8
	Yes	86	22.2
Accountant			
	No	381	98.4
	Yes	6	1.6
Pilot			
	No	385	99.5
	Yes	2	0.5
Engineer			
	No	378	97.7
	Yes	9	2.3
Freelance			
	No	371	95.9
	Yes	16	4.1
Media			
	No	386	99.7
	Yes	1	0.3
Lawyer			
	No	384	99.2
	Yes	3	0.8
Data Security			
	No	385	99.5
	Yes	2	0.5
Work online			
	No	242	62.5
	Yes	145	37.5
Part-time			
	No	327	84.5
	Yes	60	15.5
Full-time			
	No	316	81.7
	Yes	71	18.3
Unemployed			
	No	302	78
	Yes	85	22
Retired			
	No	367	94.8
	Yes	20	5.2

**Figure 1:**
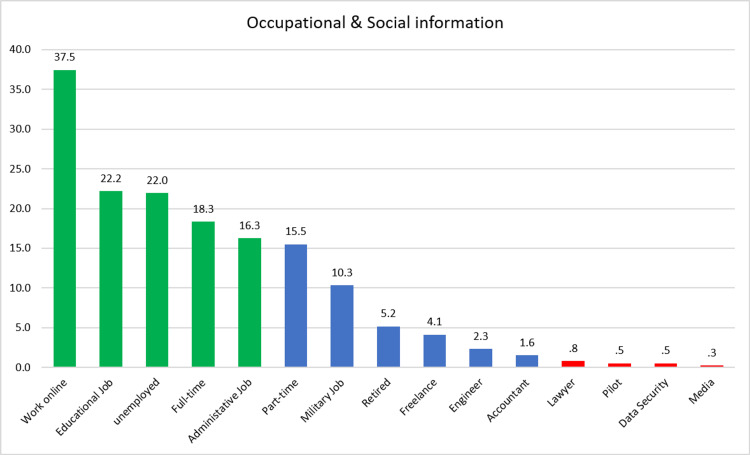
Occupational and social information of the caregivers This figure shows the number of participants based on their occupational status during the COVID-19 period

Around 145 (37.5%) of the caregivers worked online, followed by 86 (22.2%) as educators, and 63 (16.3%) had administrative jobs. Only three (0.8%) were working as lawyers, while there were only two (0.5%) pilots and data security professionals and only one caregiver working in media (Figure 1).

When the psychosocial well-being before and during was assessed on the bases of some questions, the caregivers of 100 (25.9%) children stated that they had "regular sleep habits" all the time before COVID; this decreased to 41 (10.6%) during COVID. Around 256 (66.2%) responded "it did not happen" to the question "the child had sleep disorders" before COVID; this decreased to 187 (48.3%) during COVID. For the question "the child had a poor appetite for food," the answer "most of the time" was seen in 52 (13.4%) before COVID and increased to 64 (16.5%) during COVID. When assessing for "the child had high food intake", before COVID, "all the time" was seen in 13 (3.4%), which increased to 29 (7.5%) during COVID. Around 19 (4.9%) responded that "the child eats junk food (non-nutritional)" all the time; this increased to 32 (8.3%) during COVID. All other data can be seen in Table 3.

**Table 3:** Comparison of child’s psychosocial well-being before and during COVID-19 n=Number

Children's psychosocial well-being		Pre-COVID			During COVID		
		n=387	%		n=387	%	
The child had regular sleep habits							
	It did not happen	9		2.3	56		14.5
	A few times	52		13.4	163		42.1
	Most of the time	226		58.4	127		32.8
	All the time	100		25.9	41		10.6
The child had sleep disorder							
	It did not happen	256		66.2	187		48.3
	A few times	104		26.9	129		33.3
	Most of the time	23		5.9	54		14
	All the time	4		1.0	17		4.4
The child had nightmares							
	It did not happen	241		62.3	209		54
	A few times	125		32.3	143		37
	Most of the time	19		4.9	27		7
	All the time	2		0.5	8		2.0
The child had poor appetite for food							
	It did not happen	162		41.9	156		40.3
	A few times	153		39.5	144		37.2
	Most of the time	52		13.4	64		16.5
	All the time	20		5.2	23		6
The child had high food intake							
	It did not happen	227		58.6	199		51.4
	A few times	99		25.6	102		26.4
	Most of the time	48		12.4	57		14.7
	All the time	13		3.4	29		7.5
The child eats junk food (non-nutritional)							
	It did not happen	43		11.1	70		18.1
	A few times	224		57.9	183		47.3
	Most of the time	101		26.1	102		26.3
	All the time	19		4.9	32		8.3

There was a comparison made on the child’s activity and social skills on different questions. When the caregivers were asked about whether "the child was anxious" pre-COVID, 34 (8.8%) were found to be anxious most and all of the time; during COVID, this increased to 91 (23.5%). Caregivers noticed that 22 children (5.7%) were "afraid of dying" most and all of the time before COVID, but during COVID, this increased to 53 (13.7%). Not many differences were found for the questions "child had suicidal thoughts" and "the child was hyperactive". There was a decline in the responses regarding "most of the time" and "all of the time" for "the child had many friends", "the child preferred to stay home most of the time", and the "child engages in sports" before COVID as compared to during COVID. In response to the questions about "the child watches TV" and "the child plays electronic games," the caregivers observed that "all the time" increased from 55 (14.2%) before COVID to 89 (23%) during COVID and from 94 (24.3%) before COVID to 151 (39%) during COVID, respectively (Table 4).

**Table 4:** Comparison of child’s activity and social skills before and during COVID-19 n=Number.

Children's activity and social skills		Pre COVID		During COVID	
		n=387	%	n=387	%
The child was anxious					
	It did not happen	220	56.8	160	41.4
	A few times	133	34.4	136	35.1
	Most of the time	25	6.5	65	16.8
	All the time	9	2.3	26	6.7
The child was afraid of dying					
	It did not happen	283	73.1	244	63
	A few times	82	21.2	90	23.3
	Most of the time	14	3.6	39	10.1
	All the time	8	2.1	14	3.6
The child had suicidal thoughts					
	It did not happen	367	94.8	356	92
	A few times	16	4.2	19	4.9
	Most of the time	2	0.5	4	1
	All the time	2	0.5	8	2.1
The child had difficulties concentrating					
	It did not happen	237	61.2	168	43.4
	A few times	107	27.7	142	36.7
	Most of the time	31	8	54	14
	All the time	12	3.1	23	5.9
The child was hyper-active					
	It did not happen	191	49.4	173	44.7
	A few times	125	32.3	126	32.6
	Most of the time	52	13.4	64	16.5
	All the time	19	4.9	24	6.2
The child had many friends					
	It did not happen	42	10.9	88	22.7
	A few times	148	38.2	173	44.7
	Most of the time	130	33.6	90	23.3
	All the time	67	17.3	36	9.3
The child preferred to stay home most of the time					
	It did not happen	105	27.1	91	23.5
	A few times	153	39.6	108	27.9
	Most of the time	93	24	114	29.5
	All the time	36	9.3	74	19.1
The child engages in sports					
	It did not happen	68	17.6	131	33.8
	A few times	167	43.1	157	40.6
	Most of the time	114	29.5	77	19.9
	All the time	38	9.8	22	5.7
The child watches TV					
	It did not happen	33	8.5	40	10.3
	A few times	136	35.2	113	29.2
	Most of the time	163	42.1	145	37.5
	All the time	55	14.2	89	23
The child plays electronic games					
	It did not happen	35	9.1	33	8.5
	A few times	122	31.5	68	17.6
	Most of the time	136	35.1	135	34.9
	All the time	94	24.3	151	39

**Figure 2:**
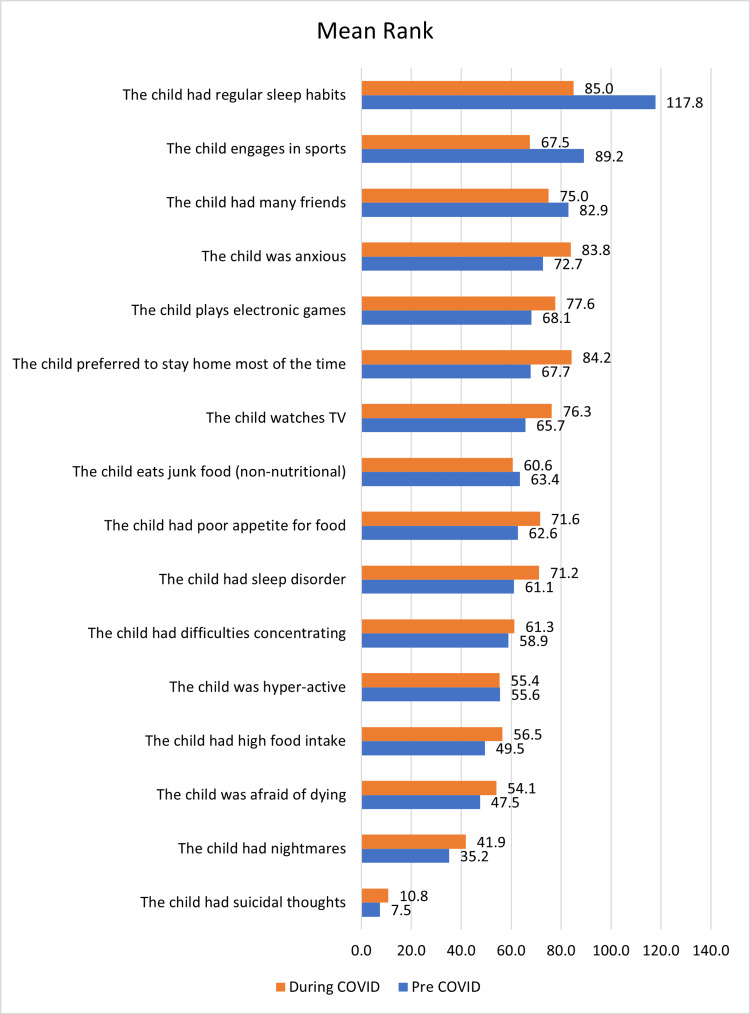
Comparison of children’s psychosocial well-being before and during COVID-19 The numbers in this figure represent the mean rank of responses for certain questions.

The following statements, "the child had regular sleep habits," "the child was hyper-active", "the child had many friends", and "the child engages in sports" had higher median scores before COVID as compared to the scores during the COVID lockdown, which was a significant difference (p <0.05). "The child had a sleep disorder," "the child had nightmares", "the child had high food intake", "the child was anxious", "the child was afraid of dying," ‘the child had suicidal thoughts," "the child had difficulties concentrating," "the child preferred to stay home most of the time", "the child engages in sports", "the child watches TV" and "the child plays electronic games" had lower median scores before COVID as compared to during COVID with significant p-value (<0.05). For the questions "the child had a poor appetite for food" and ‘the child eats junk food (non-nutritional),’ there was no significant mean difference before and during COVID (p>0.05) (Table 5, Figure 2).

**Table 5:** Comparison of children’s psychosocial well-being before and during COVID-19 IQR: interquartile range; pre: pre-COVID; during: during COVID

Children’s psychosocial well-being and lifestyle (n=387)	Median	IQR		Mean Rank	Z	p
The child had regular sleep habits (pre)	2	2	3	117.8	-11.29	<0.001
The child had regular sleep habits (during)	1	1	2	85.0		
The child had sleep disorder (pre)	0	0	1	61.1	-7.63	<0.001
The child had sleep disorder (during)	1	0	1	71.2		
The child had nightmares (pre)	0	0	1	35.2	-4.26	<0.001
The child had nightmares (during)	0	0	1	41.9		
The child had poor appetite for food (pre)	1	0	1	62.6	-1.68	0.092
The child had poor appetite for food (during)	1	0	1	71.6		
The child had high food intake (pre)	0	0	1	49.5	-4.54	<0.001
The child had high food intake (during)	0	0	1	56.5		
The child eats junk food (non-nutritional) (pre)	1	1	2	63.4	-0.15	0.879
The child eats junk food (non-nutritional) (during)	1	1	2	60.6		
The child was anxious (pre)	0	0	1	72.7	-7.27	<0.001
The child was anxious (during)	1	0	1	83.8		
The child was afraid of dying (pre)	0	0	1	47.5	-5.29	<0.001
The child was afraid of dying (during)	0	0	1	54.1		
The child had suicidal thoughts (pre)	0	0	0	7.5	-3.50	<0.001
The child had suicidal thoughts (during)	0	0	0	10.8		
The child had difficulties concentrating (pre)	0	0	1	58.9	-7.58	<0.001
The child had difficulties concentrating (during)	1	0	1	61.3		
The child was hyper-active (pre)	1	0	1	55.6	-2.84	0.004
The child was hyper-active (during)	1	0	1	55.4		
The child had many friends (pre)	2	1	2	82.9	-7.76	<0.001
The child had many friends (during)	1	1	2	75.0		
The child preferred to stay home most of the time (pre)	1	0	2	67.7	-5.86	<0.001
The child preferred to stay home most of the time (during)	1	1	2	84.2		
The child engages in sports (pre)	1	1	2	89.2	-7.26	<0.001
The child engages in sports (during)	1	0	2	67.5		
The child watches TV (pre)	2	1	2	65.7	-2.89	0.004
The child watches TV(during)	2	1	2	76.3		
The child plays electronic games(pre)	2	1	2	68.1	-6.78	<0.001
The child plays electronic games(during)	2	1	3	77.6		

There was a comparison made to assess the difference among gender for children’s psychosocial well-being and lifestyle. For the questions asking "The child had high food intake", "the child eats junk food (non-nutritional)", although males had a higher mean rank in the pre-COVID period, it was insignificant. However, it turned out to be significant during the COVID era, with males having higher mean rank than females (p=0.022), and (p=0.015), respectively. "The child was hyperactive", "the child preferred to stay home most of the time", and "the child plays electronic games", males had higher mean ranks in all queries compared to females in both pre-COVID and during COVID time with p<0.05. In all other questions, there was no significant difference in gender (Table 6).

**Table 6:** Comparison among genders of children’s psychosocial well-being before and during COVID-19 p: p-value

Children’s psychosocial well-being and lifestyle		Pre-COVID		During COVID	
		Mean Rank	p	Mean Rank	p
The child had regular sleep habits	Male	196.2	0.581	191.9	0.627
	Female	190.6		197.2	
The child had sleep disorder	Male	193.8	0.952	192.0	0.638
	Female	194.3		197.0	
The child had nightmares	Male	195.9	0.619	193.4	0.890
	Female	191.0		194.9	
The child had poor appetite for food	Male	194.3	0.942	189.7	0.322
	Female	193.5		200.5	
The child had high food intake	Male	200.3	0.121	203.6	0.022
	Female	184.4		179.3	
The child eats junk food (non-nutritional)	Male	202.0	0.051	204.4	0.015
	Female	181.8		178.1	
The child was anxious	Male	191.8	0.581	189.5	0.295
	Female	197.4		200.9	
The child was afraid of dying	Male	189.5	0.208	186.7	0.063
	Female	200.9		205.2	
The child had suicidal thoughts	Male	193.0	0.586	193.3	0.736
	Female	195.5		195.1	
The child had difficulties concentrating	Male	195.5	0.712	195.1	0.800
	Female	191.8		192.3	
The child was hyper-active	Male	208.5	0.001	209.3	<0.001
	Female	171.8		170.6	
The child had many friends	Male	189.8	0.336	191.6	0.576
	Female	200.4		197.7	
The child preferred to stay home most of the time	Male	202.9	0.042	204.4	0.020
	Female	180.4		178.1	
The child engages in sports	Male	196.4	0.582	193.6	0.922
	Female	190.3		194.6	
The child watches TV	Male	197.8	0.374	195.3	0.764
	Female	188.1		192.0	
The child plays electronic games	Male	204.3	0.019	206.7	0.003
	Female	178.3		174.6	

## Discussion

This cross-sectional study analyzed the data regarding the impact of COVID-19 lockdown time on children's psychosocial well-being. The study's demographic information reveals several significant findings. The majority of caregivers were mothers, which is consistent with traditional gender roles and societal expectations, also the ideology of maternal altruism which stipulates that mothers are more naturally predisposed to nurturing and self-sacrifice. Half of the caregivers' children belonged to the 1-6 years age group, indicating that younger children require more care and attention from their parents or guardians. It is positive to note that a significant proportion of caregivers work online, providing greater flexibility in balancing work and caregiving responsibilities.

A worsening of sleep habits and sleep disorders among children was observed probably due to poor mental health, increased use of electronics, and lack of a regular schedule. Similar data was also reported regarding sleep quality in children [11]. Furthermore, a cross-sectional study conducted after an extensive web-based Italian survey mentioned that factors such as household economic concerns, household food insecurity, and parents' perception regarding the increased difficulty of the family means after the pandemic were associated with children's sleep disorders [12].

In addition, our results shed light on eating habits; children's food and junk food consumption showed an increase during COVID-19 compared to before the pandemic. A previous local study also reported that the COVID-19 pandemic negatively impacted children's eating habits due to decreased levels of activity and changes in food intake [13]. Furthermore, COVID-19 was a significant reason for fast food consumption and sedentary time during the lockdown [14]. Reasons for that could be due to child boredom, trying to stimulate their taste buds with junk food, trying to kill time during the lockdown, or due to bad parental habits of ordering fast food to try to improve their children's mood during the pandemic, which was filled with negativity and fear. Moreover, there is a relationship between junk food consumption and psychological distress, establishing this as one of the reasons for the increased anxiety among children other than COVID-19 [15].

Also, mental health issues such as anxiety and fear of death increased during the pandemic due to the negative news associated with COVID-19 and the many deaths observed and caused by this pandemic. In addition, financial insecurity during COVID-19 led to unhealthy parenting practices, further affecting children's well-being and mental health. Also, there is a relation between less sleep and anxiety, as children with lower sleep duration reported higher anxiety levels. Furthermore, children who had higher durations of screen time during COVID-19 also had higher levels of anxiety and depressive symptoms, as explained in [16].

Moreover, an increase in screen time was observed during COVID-19 through watching TV and playing video games. This adversely affected the children's cognitive function and attention span and also exacerbated aggressive behaviors. Furthermore, a study showed that video games are able to influence children who have attention problems, leading to lack of self-control and impulsiveness [17]. Also, greater screen time could be related to a higher intake of food through binge eating [18]. When it came to children's suicidal thoughts, there has not been a noticeable difference in the two periods we targeted, which could be attributed to factors such as a sense of loyalty to their parents where children have been noticed to hide these feelings from them [19]. As such, there can be a bias, because parents' observation may overshadow an existing problem. Our survey showed a decrease in children's friendships during the pandemic and quarantine period. Even though virtual contact could still be established between children and their friends, the physical aspect remained important in social development [20]. We also observed that there was an increase in child's preference to stay at home during the pandemic. This point, in particular, can be evaluated from several elements, as it was noticed that the pandemic contributed to increasing overall anxiety for children [21]. Nevertheless, other research pointed out that it had a hand in strengthening the bonds between parents and their kids [22]. Additionally, children's participation in sports activities lessened. This can be attributed to a decreased ability to engage in outdoor activities. The sudden transition has been proven to affect children's physical activity, which led to more sedentary behavior [23].

Regarding the limitations of this study, the findings are limited to the research sample. Another limitation is that the electronic questionnaire was distributed through social media, which may lower the reliability of the responses provided by the participants. Another is that many cities in Saudi Arabia have dealt with the COVID-19 pandemic differently, especially during the lockdown period. As such, we recommend a more thorough and detailed investigation of the leftover consequences of the pandemic, focusing on the long-term effects of the COVID-19 pandemic on children's psychosocial well-being.

## Conclusions

The COVID-19 quarantine period has had a negative impact on children's psychosocial well-being, with observed worsening of sleep habits and sleep disorders, increased junk food consumption, and higher levels of anxiety and depressive symptoms. In addition, the decreased social interaction with friends during the quarantine period further adds to children's challenges during this pandemic. It is recommended to implement actions that aim to enhance the ability of children to cope with challenges by improving communication to address their worries and apprehensions, promoting regular physical activity and routines, and implementing measures to mitigate feelings of isolation.
